# ER stress as a trigger of UPR and ER-phagy in cancer growth and spread

**DOI:** 10.3389/fonc.2022.997235

**Published:** 2022-11-03

**Authors:** Alessandro Cherubini, Ester Zito

**Affiliations:** ^1^ Istituto di Ricerche Farmacologiche Mario Negri IRCCS, Milan, Italy; ^2^ Department of Biomolecular Sciences, University of Urbino Carlo Bo, Urbino, Italy

**Keywords:** ER-phagy, UPR, ERO1 alpha, ER stress, hypoxia, cancer

## Abstract

Tumors can survive environmental and metabolic stress by triggering homeostatic responses that re-establish the pre-stress status and permit them to grow and thrive. The endoplasmic reticulum (ER) is the organelle where proteins undergo post-translational modifications and are folded and exported to the secretory pathway. Its environment and activity are therefore fundamental for proteostasis, i.e., the plethora of mechanisms controlling protein formation, folding, degradation, and secretion, needed to assure protein balance and cellular health. In different tumor-related conditions, such as after the activation of oncogenes or under hypoxia and nutrient deprivation, the ER experiences stress, triggered by a high load of proteins to be folded compared to the limited folding capacity of the organelle. As a consequence, three ER membrane sensors and the related unfolded protein response (UPR) are activated. The UPR comprises a complex interconnection between signal transduction pathways that promote a homeostatic response that acts by increasing the amount of protein chaperones and of proteins involved in ER-associated protein degradation (ERAD) on one hand and attenuating protein translation on the other. ER-phagy, literally “eating” the ER, is part of another homeostatic response consisting of the clearance of non-functional ER portions including misfolded proteins. This response is also activated by a set of dedicated ER-phagy receptors after ER stimuli, which overlap the stimuli generating ER stress. Thus, the UPR and ER-phagy are two closely related homeostatic mechanisms that cooperate in re-establishing ER homeostasis. However, while the role of the UPR in favoring cancer growth and thriving by promoting angiogenesis, metastasis, chemotherapy resistance, and epithelial-to-mesenchymal transition is consolidated, that of ER-phagy is still in its infancy. This essay provides an overview of emerging concepts on ER stress, the UPR, and ER-phagy and their crosstalk in tumorigenesis. We also critically review new findings on their pharmacological targeting in cancer.

## Introduction

The stress of the endoplasmic reticulum (ER stress), as the term suggests, is a condition of the stress of this organelle, which is central in protein folding and secretion. Thus, the endoplasmic reticulum (ER) suffers this condition when its folding ability is impaired in the face of a high load of proteins. A number of intrinsic and extrinsic factors are the sources of ER stress in tumors. As intrinsic factors, there are oncogenes (K-Ras and c-MYC) or the loss of tumor suppressors (e.g., p53), which initiate neoplastic transformation by driving rapid and uncontrolled cell proliferation and are thus associated with an increased protein translation, causing ER stress (1, 2). As extrinsic factors, tumors, especially solid ones, deal with a characteristic microenvironment characterized by hypoxia, the shortage of nutrients, and high concentrations of some metabolites (e.g., lactate and fatty acids) (3). Hypoxia, for example, which is the trigger of angiogenesis, is also a trigger of ER stress as it impairs the post-translational disulfide bond formation of proteins in the ER (4). Thus, hypoxia in tumors might be considered a constant source of ER stress.

In cancer cells, the initial nutrient deprivation of glucose and glutamine limits intermediary metabolism and the hexosamine biosynthetic pathway (HBP), which generates substrates for N-linked protein glycosylation that are important for protein folding, triggering ER stress in cancer (5). ER stress activates the homeostatic UPR through the stimulation of the three ER stress sensors IRE1, PERK, and ATF6, which articulate a sophisticated multifaceted response by upregulating enzymes dedicated to the protein folding machinery and degradation on one side and attenuating protein translation on the other, finally re-establishing ER homeostasis (6). Thus, the UPR exerts a cytoprotective effect on tumor cells and promotes tumor progression and spread by inducing different mechanisms involved in the thriving of cancer cells, such as angiogenesis, epithelial–mesenchymal transition (EMT), and resistance to chemotherapy (7, 8).

In tumors, the same ER stimuli–inducing ER stress also triggers ER-phagy. Despite the initial idea of ER-phagy, like the uncontrolled eater of the ER, the discovery of specific ER-phagy receptors, whose number is in rapid expansion (FAM134A, FAM134B, FAM134B-2, FAM134C, SEC62, RTN3L, CCPG1, ATL3, and TEX264), has redrawn the initial picture favoring the hypothesis of a selective ER-induced mechanism (9). ER-phagy is activated by the stimulation of various receptors and involves breaking down “malfunctioning” ER portions by sequestering them in autophagosomes and promoting the resulting degradation of the cargo through fusion with lysosome (10). Thus, ER-phagy may lead the cells to reshape the ER and thus survive severe ER stress. Ample evidence suggests that the UPR and ER-phagy are two closely interconnected homeostatic processes that may complement each other and both aim at re-establishing ER homeostasis and help cells thrive.

The study of ER-phagy is more recent than the UPR and gained momentum in the last 10 years with the discovery of its dedicated receptors. However, its involvement in tumorigenesis is still controversial and it is debated whether it is a pro-survival or a pro-death response.

## Endoplasmic reticulum stress and unfolded protein response

**Figure 1:**
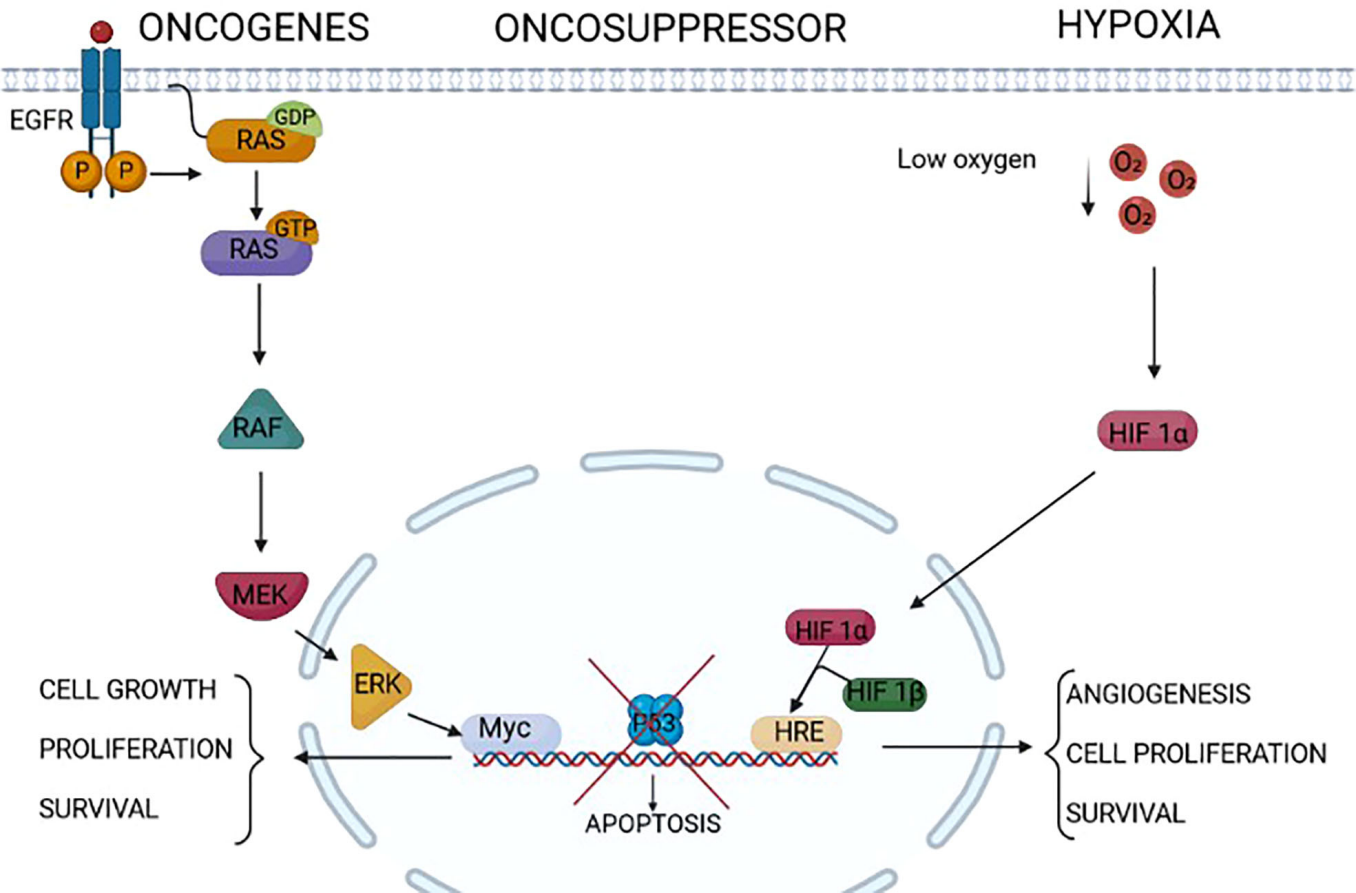
Endoplasmic reticulum (ER) stressors in cancer. Different factors/conditions related to cancer are considered ER stressors. The epidermal growth factor receptor family, composed of four members HER1, HER2, HER3, and HER4, is highly expressed in some cancers (for example, HER1/HER2 in gastric cancer and HER2 in breast cancer). The activation of HER2 and HER1 results in the activation of intracellular pathways including RAS/RAF/MEK/ERK, PI3K/AKT/TOR, Src family kinases, and STAT transcription factors that modulate survival, proliferation, mobility, and cancer cell invasiveness. Some tumors express RAS mutations that activate downstream signal transduction independently from upstream receptor activation. The increased cell proliferation relies on increased protein translation that is a source of ER stress in these tumors. On the same line, the lack of the activity of oncosuppressors such as p53 triggers uncontrolled cell proliferation and growth, which might induce ER stress. Extrinsic factors, i.e., independent from the genetic makeup of the tumor, as a low concentration of oxygen, hypoxia, might induce ER stress in tumors. Indeed, this is a common condition of solid tumors, which not only leads to the assembly of the two components HIF1alpha and HIF1beta of HIF1, rendering it an active transcription factor of genes involved in angiogenesis and cell proliferation, but also impairs the formation of post-translational disulfide bonds in proteins, triggering ER stress.

The ER is a cellular organelle where important protein modifications take place such as oxidative protein folding and N-glycosylation, thereby regulating protein trafficking and secretion; it is also the main site of intracellular calcium storage and for the control of lipid homeostasis (11). Thus, this organelle suffers stress when some processes in protein folding or degradation become faulty or inefficient and the load of unfolded protein accumulates beyond a tolerable threshold (12). As a consequence, an expansion of the ER membrane takes place, which is driven by lipid biosynthesis and alleviates this stress by leading the accommodation of an increased amount of ER client proteins and limiting their aggregation independently from the chaperone levels (13). Characteristic tumor conditions such as nutrient deprivation and hypoxia, which are common in solid cancers and are also associated with their aggressiveness (3), impair protein folding, triggering ER stress (**Figure 1**) (4). The ER stress-induced UPR constitutes a plethora of corrective measures initiated by three ER stress sensors: protein kinase R-like ER kinase (PERK), inositol-requiring enzyme 1 (IRE1), and activating transcription factor 6 (ATF6) which promote the inhibition of protein translation and antioxidant response by the PERK branch together with increased chaperone activity, lipid biosynthesis, and protein degradation by the concerted IRE1 and ATF6 branches to restore ER homeostasis in the first instance (6). However, persistent unresolved ER stress, together with conditions favoring the maladaptive branch of the UPR, result in cell death, revealing the double-edged sword of UPR as a pro-death rather than pro-survival response (6).

## The three unfolded protein response sensors

IRE1 is the most conserved of the three sensors, already present in the simple eukaryote yeast. It is a type I ER transmembrane protein whose cytoplasmic part contains an auto-phosphorylating kinase domain and an endoribonuclease (RNase) one. In the conditions of ER stress, the luminal domains of two IRE1 protomers dimerize, leading to the autophosphorylation of the protein and the activation of the RNase domain. The latter promotes the unconventional splicing of a 26-bp intronic region of the X box binding protein 1 mRNA (*XBP1*), which, after being translated and translocated in the nucleus, becomes a transcription factor of the genes involved in protein folding and in ER-associated protein degradation (ERAD) (14).

PERK and ATF6 appear in metazoans rendering the UPR a more complex and articulated signal transduction in superior eukaryotes. PERK is a type I transmembrane protein of the ER that, after ER stress, undergoes the dimerization and autophosphorylation of the cytosolic domain, which then promotes the phosphorylation of the eukaryotic initiation factor 2-alpha (eIF2-alpha), limiting protein translation, and the phosphorylation of NRF2, activating an antioxidant NRF2-dependent response (14–16).

On the other hand, the activation of PERK promotes the selective translation of ATF4, a transcription factor that triggers the expression of stress-responsive genes such as the transcription factor C/EBP homologous protein (CHOP), with the downstream ER protein disulfide oxidase, ERO1 alpha (henceforth, ERO1), and the phosphatase of P-eIF2-alpha, GADD34, which reactivates protein translation (17–19).

**Figure 2:**
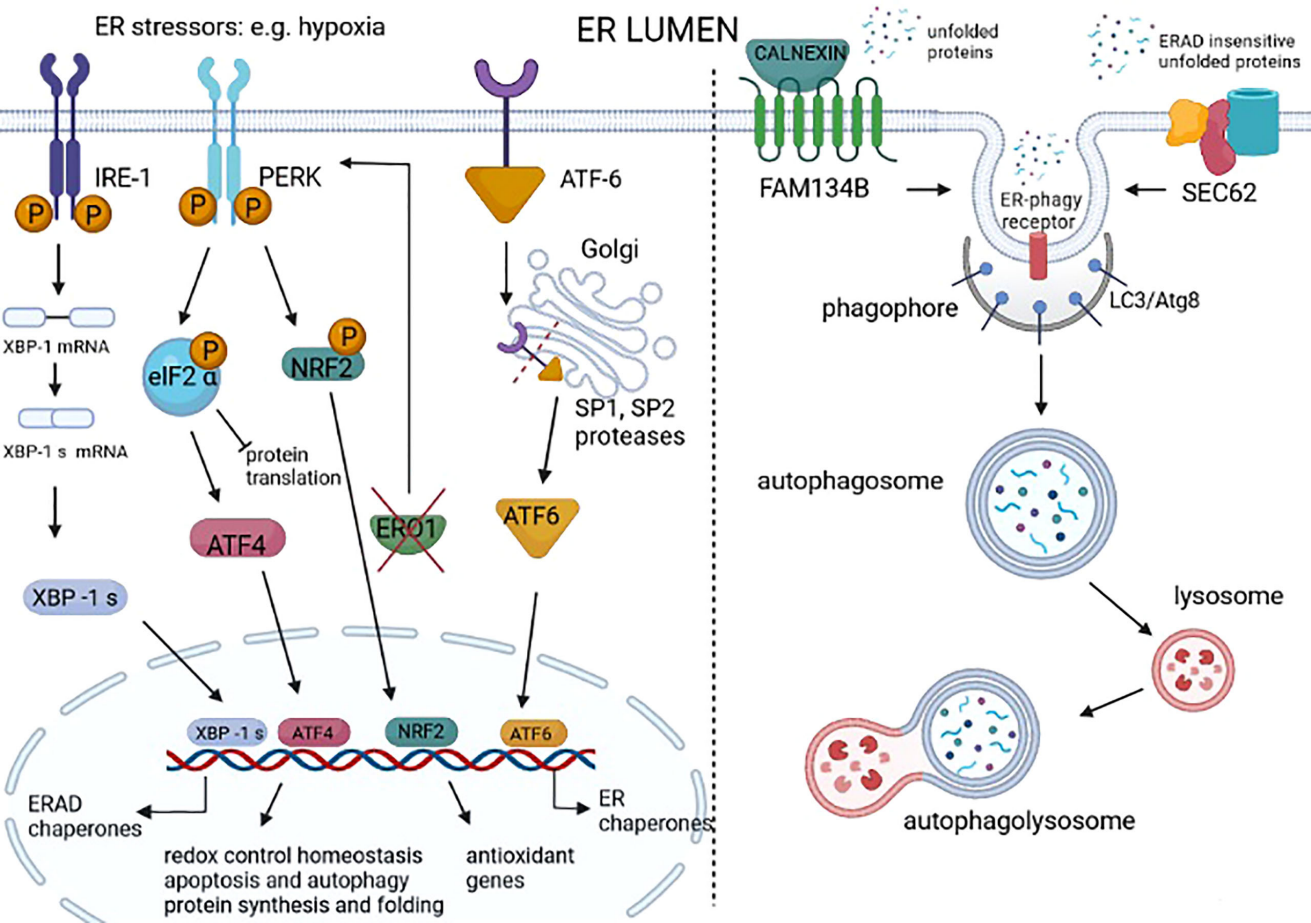
Unfolded protein response (UPR) and ER-phagy in cancer. UPR is a homeostatic response to ER stress that is present in many cancer types. UPR is activated by three different sensors on the ER membrane: IRE1, PERK, and ATF6. IRE1 dimerizes following ER stress, activating an RNAse domain that promotes the unconventional splicing of XBP1 mRNA (XBP1s). The translated XBP1s acts as a transcription factor of genes involved in ER-associated degradation (ERAD) and chaperones. ER stress–activated PERK phosphorylates eIF2 alpha, promoting the attenuation of protein translation while also promoting the phosphorylation of NRF2, thereby the transcription of genes with an antioxidant function. The PERK signal also favors the selective translation of ATF4, which regulates the redox control, the genes involved in autophagy, and the CHOP-ERO1 axis. Regarding the axis CHOP-ERO1, we have seen that in breast cancer cells under hypoxic conditions, ERO1 is not downstream to CHOP. However, the lack of ERO1 converges and activates the PERK signal (21). ATF6 translocates in the Golgi where it is cleaved by SP1 and SP2 proteases and acts as a transcription factor of ER chaperones. ER stress also activates ER-phagy, a mechanism that leads to the clearance of ER portions containing misfolded proteins. FAM134B is an ER-phagy receptor that, through a physical interaction with a protein adaptor, such as calnexin, might sense unfolded proteins and starts the autophagy of the ER. SEC62 is another ER-phagy receptor that leads to the ER-phagy of ER portions containing ERAD-insensitive unfolded proteins.

ATF6 is a type-II ER transmembrane protein whose ER stress–mediated activation leads to its trafficking to the Golgi, where the cytosolic part is cleaved from the transmembrane domain by SP1 and SP2 proteases. Its free cytosolic portion can then migrate to the nucleus and act as a transcription factor of protein chaperones such as BIP/GRP78 and GRP94 (20) (**Figure 2**).

Interestingly, the UPR is not only activated by unfolded proteins into the ER but also by an abnormal/excessive saturation of membrane lipids (22, 23). This occurs because the transmembrane domain of UPR sensors senses the surrounding membrane composition, and not only unfolded ER proteins. The IRE1/XBP1s branch of the UPR is a well-characterized positive regulator of lipid biosynthesis: indeed, XBP1s triggers ER expansion together with the regulation of lipogenic genes such as *Dgat2*, *Scd1*, and *Acc2* and increased lipid biosynthesis (24, 25). To conclude, the UPR or, to better say, the ER stress response is activated by both unfolded proteins and excessive saturated fatty acid which give rise to a lipotoxic stress.

As mentioned before, the UPR is mainly a homeostatic response whose primary goal is to relieve cells from stress by enacting corrective measures, to re-establish ER homeostasis. However, chronic unresolved ER stress together with the activation of maladaptive UPR factors in specific genetic and environmental contexts may trigger cell death (6).

## Unfolded protein response in cancer

The UPR is present in many type of cancers, such as breast, pancreas, lung, skin, prostate, brain, and even liquid cancer (i.e., leukemia and lymphoma) (26) and play a role in cancer-related processes that favor cancer thrive and metastasis.

For example, as regards tumor angiogenesis, i.e., the formation of new blood vessels, oxygen levels can fall to 0.01% in tumor cells and under such low-oxygen tension, the two HIF-1 components alpha and beta are assembled, promoting the transcription of angiogenic factors and hence angiogenesis (27). Angiogenesis feeds the tumor by delivering nutrients and oxygen and removing the waste products of the aerobic metabolism (28).

Furthermore, newly formed vessels, i.e., *de novo* angiogenesis, increase the cells’ ability to spread from the primary tumor throughout the body, forming metastatic niches. Thus, angiogenesis correlates with tumor aggressiveness by leading the formation of tumors with high metastatic potential (29).

The inhibition of all three UPR sensors, PERK, IRE1 alpha (henceforth, IRE1), and ATF6 limits cancer angiogenesis (30) (31) (32). Downstream from PERK, ATF4 promotes a direct transcription of the angiogenic master regulator vascular endothelial growth factor A (VEGFA) (31). ERO1 is a protein disulfide oxidase that participates to the process of the disulfide bond formation of the new nascent protein of the ER. ERO1 is strongly induced in hypoxia (33), and is highly expressed in the most aggressive basal breast cancer, and analysis from the Metastatic Breast Cancer project indicates an inverse relation between ERO1 levels in the primary aggressive triple-negative breast cancer (TNBC) and the time at which distant metastases are detected, arguing for a pivotal role of ERO1 in the aggressive metastatic breast cancer phenotype (34).

Recent secretomic analyses indicate that the lack of ERO1 in breast cancer cells has no major impact in normoxia but impairs different angiogenic factors in hypoxia, suggesting a selective effect on angiogenesis. At the molecular level, the lack of ERO1 not only impairs the intermolecular disulfide bonds of VEGFA but also promotes a hyper-N-glycosylation of the isoform VEGF^121^ that blunts its secretion (35). This explains why ERO1-deficient TNBC xenografts have decreased vasculogenesis, together with a reduced ability to generate lung metastases, suggesting that ERO1 inhibition might be a good tool to selectively impair angiogenesis in solid tumors and limit metastasis (34). The detrimental effect of ERO1 loss on tumor angiogenesis following impairment in the secretion of angiogenic factors also potentiates the cytotoxic effect of VEGF-target antiangiogenic therapy in breast tumor xenografts, which is not particularly effective as single therapy (34).

Gene expression analysis in breast cancer patients supports a more positive outcome in terms of overall survival when the ratio between the levels of PERK and ERO1 is high, indicating some kind of cooperation between these two in breast tumor growth and spread (21).

While ERO1 activity as protein disulfide oxidase is well compensated by other ER oxidases in normoxic conditions, its lack predisposes to proteotoxicity in hypoxic conditions as manifested by the accumulation of detergent-insoluble protein aggregates. As a consequence, ERO1 loss activates the PERK branch in hypoxic conditions in TNBC, thus repressing protein translation, quite likely as an adaptive pro-survival mechanism of cells that lack an enzyme involved in protein folding, and are thus susceptible to impaired proteostasis (**Figure 2**).

The forced restart of protein translation by the inhibitor of the integrated stress response (ISRIB), which limits the activity of P-eiF2 alpha, triggers proteotoxicity and death in ERO1-devoid TNBC xenografts. This suggests that of some tumors ERO1 loss or inhibition might enhance the cytotoxic response to some drugs that promote the restart of protein translation when proteostasis is impaired (21).

Like in tumor angiogenesis, the UPR is also involved in EMT, which is the switching of cells from an epithelial phenotype with adhesion properties to a mesenchymal one. EMT leads to the loss of contacts between cells and upregulates extracellular matrix proteins, facilitating the migration and invasion of cancer cells. The branch PERK-eiF2 alpha was upregulated in tumor cells expressing EMT markers and is required for malignancy (36). Furthermore, the small molecule 4μ8c, which blocks the IRE1-alpha signal, hinders EMT, suggesting that both PERK and IRE1-alpha might be involved in cancer EMT (37).

Targeting ATF6 in dormant tumor cells prolonged the survival of dormant tumor cell–bearing nude mice, suggesting that the ATF6 signal was involved in acquiring a dormant phenotype of tumor cells, which causes cancer recrudescence (38).

Other tumor-related conditions in which the UPR is involved are related to chemotherapy resistance and consequent tumor relapse. The taxane drug paclitaxel is one of the first-line chemotherapies in breast tumors. Its cytotoxic effect is due to the ability to be an inhibitor of the spindle formation and is dependent on the cells’ capacity to divide. A paclitaxel-induced UPR is well documented in breast tumors and in TNBC (7, 39, 40) and the consequent UPR induction is considered among the reasons for the chemotherapy resistance and tumor relapse. Paclitaxel enhances IRE1 RNase activity and contributes to the tumor relapse in a xenograft mouse model of TNBC (40). The ERO1-devoid xenograft mouse model of TNBC grows slowly, and so, the cells also divide slowly. This might explain the lower response of ERO1-devoid TNBC to paclitaxel. However paclitaxel-treated ERO1-devoid TNBC blunts the UPR suggesting that the lack of ERO1 might contribute to the inhibition of tumor relapse and chemotherapy resistance in these tumors by attenuating the UPR (21).

The knockdown of PERK promotes the survival of luminal breast cancer cells treated with the combination of lapatinib (a tyrosine kinase inhibitor) and obatoclax (a pro-survival BCL-2 family inhibitor) by reducing autophagy (41). In contrast, ATF4, downstream to PERK, was important in mediating a pan-peptidylarginase deiminase to kill TNBC cells through the activation of mTOR signaling and the enhancement of autophagy (42). This suggests a double role of PERK and its signaling in both promoting and inhibiting autophagy, with consequent reduced and increased tumor survival, respectively.

## Targeting endoplasmic reticulum stress and unfolded protein response in cancer

Some drugs might push ER stress in cancer cells beyond their tolerance threshold and trigger apoptosis, exploiting the chronic ER stress as a tumor weakness. For example, this is the case of celecoxib, a COX-2 specific non-steroidal anti-inflammatory drug approved by the US Food and Drug Administration (FDA) for treating pain and inflammation, which leads cancer cells to death by potentiating its intrinsic ER stress (43).

On the basis of the same rationale, bortezomib (BTZ), a dipeptidyl boronic acid, is used to fight some tumors with highly secretory features such as hematologic malignancies. BTZ was approved by the FDA in 2003, for the treatment of relapsed/refractory multiple myeloma (MM), in 2006, for the treatment of refractory/relapsed mantle cell lymphoma, and, in 2014, for previously untreated mantle cell lymphoma (44).

BTZ is a reversible inhibitor of the 26S proteasome, which is part of the ubiquitin proteasome pathway and a central player in ERAD, so it inhibits misfolded protein degradation, resulting in a consequent increased accumulation of misfolded protein and thus proteotoxicity in highly secretory cells. Its cytotoxic effect in MM is directly related to the conspicuous amount of immunoglobulin retained inside MM cells and thus to their highly secretory phenotype, which predisposes them to the proteotoxicity. Indeed, there is a good correlation between the secretory cell phenotype of some hematological malignancies and the BTZ cytotoxic effect: the suppression of XBP1s facilitates resistance to the cytotoxic effect of proteosome inhibitors in the progenitors of plasmablasts by inhibiting plasma cell maturation and hence immunoglobulin production (45).

Sunitinib is an orally delivered tyrosine kinase inhibitor that is FDA-approved for the treatment of metastatic renal cell carcinoma. By virtue of its activity as a kinase inhibitor, it also inhibits IRE1 kinase activity (46).

An array of drugs that selectively inhibit the signal transductions of the three sensors of the ER stress response (UPR) are also now available.

A first-in-class PERK inhibitor, GSK2656157, was selective for the PERK inhibition of multiple human tumor xenograft growth in mice by impairing angiogenesis. However, this inhibitor is associated with a serious side effect due to the inhibition of PERK activity in the pancreas, the so-called effect on the target off-tumor, which leads to impaired pancreatic function (47). Studies on GSK2656157 observed that the molecule repressed TNF-mediated RIPK1 kinase-dependent death in a PERK-independent manner, suggesting a potential off-target effect of this inhibitor that had instead previously been considered a highly selective PERK inhibitor (48).

MKC8866 is a small-molecule IRE1 RNase inhibitor, which was first described by Patterson and colleagues in 2011 (49). In a xenograft mouse model of TNBC, MKC8866 increases paclitaxel-mediated tumor suppression and reduces tumor relapse after therapy with taxane, suggesting that it can render chemotherapy with taxane more effectively, limiting tumor relapse (40).

Currently, given the off-target effects, the only UPR modulator that entered clinical testing is MKC-8866 (ORIN1001), which is tested in breast cancer patients with advanced tumors in combination with taxanes (https://clinicaltrials.gov/ct2/show/NCT03950570).

Among the small-molecule-type UPR modulators that have a good safety profile and are still not EMA/FDA approved, ISRIB is worth being mentioned. ISRIB enhances the guanine nucleotide exchange factor (GEF) activity of eIF2B, generating GTP, which is one of the three components of the ternary complex required to initiate protein translation. Thus, eIF2B becomes resistant to the inhibitory effect of p-eIF2alpha and reactivates the repression of the protein translation, downstream to the PERK signal transduction. At the moment, ISRIB has not shown any off-target effects and its good safety profile in preclinical cancer models suggests the possibility of using it in humans (50–55).

**Figure 3:**
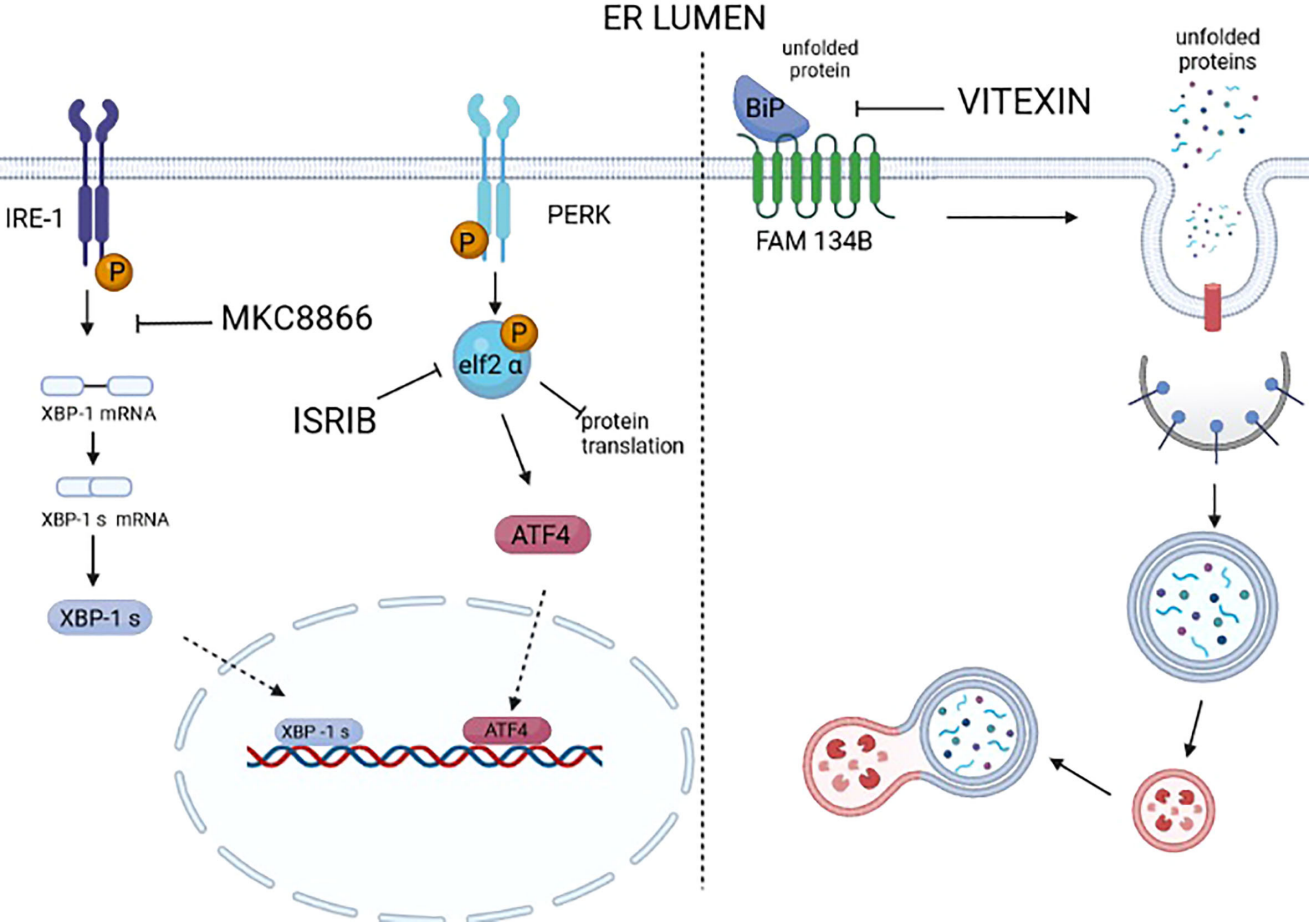
Drugs targeting UPR and ER-phagy. Many selective modulators or inhibitors of UPR sensors and downstream mediators are available. However, a large number of these modulators/inhibitors fail to enter clinical practice because of off-target effects. At the moment, only MKC8866, an inhibitor of the RNAse activity of IRE1, is under clinical trial. It is worth mentioning that ISRIB, an inhibitor of the activity of P-eIF2 alpha in repressing protein translation, thus reactivates protein translation. ISRIB has a good safety profile in the preclinical models of cancer; at the moment no off-target effects are reported, and it was proven to be a valid drug in counteracting prostate cancer, KRAS-positive lung xenografts, hypoxic breast xenografts, and ERO1-devoid TNBC xenografts. We still lack the selective inhibitors/modulators of ER-phagy. Recently, vitexin, a plant-derived flavone O-glycoside, was shown to disrupt the complex FAM134B-BIP and inhibit breast cancer (MCF7-derived) xenografts.

ISRIB was effective on chemotherapy-resistant KRAS mutant lung cancer with high PERK/p-eIF2alpha (56), on hypoxic breast tumors (56) and on TNBC devoid of ERO1 (21). We detected an important cytotoxic effect in ERO1-devoid TNBC xenograft-bearing mice treated with the combination of paclitaxel and ISRIB, suggesting that the proteotoxic effect due to the loss of the ER oxidase ERO1 predisposes cells to the cytotoxic effect of the ISRIB-dependent restart of protein synthesis in breast cancer (**Figure 3**).

To conclude, the dual effect of ER stress in generating cell death, if it is excessive, or UPR-mediated cell survival, if moderate, may undermine the rationale of ER stress/UPR-targeting drugs in cancer and calls for careful analysis before starting targeting these two mechanisms for the pharmacological treatment of cancer.

## ER-phagy

In 2006, a seminal paper from Peter Walter’s laboratory revealed, through electron microscopy analysis, the presence of ER membranes in autophagosome-like structures. They noticed that after treatment with the reducing agent DTT, which, by inhibiting disulfide bond formation, induces ER stress and the UPR, yeast developed autophagosomes containing the membrane stacks derived from the UPR-expanded ER (57) (58). This suggested that ER sequestration controls ER size and thus might represent a way to reach a new steady state in an ER that is engulfed together with unfolded proteins.

Subsequent studies suggested that autophagy of the ER, later called ER-phagy, is also active in cells in basal conditions but increases under ER stress stimuli (10), also suggesting a role of ER-phagy in basal conditions.

ER-phagy operates on both the reshaping of ER expansion following ER stress and the lysosomal degradation of protein aggregates in the ER lumen. ER-phagy can be mediated by autophagosome (macro-ER-phagy) or independently from autophagosomes (micro-ER-phagy) through the direct engulfment of ER fragments by the endolysosomal system (59). For a long time, autophagy was thought of as a non-selective pathway that randomly degrades parts of organelles. However, the selectivity and the fine-tuned regulation of macro-ER-phagy (henceforth ER-phagy) was recently appreciated following the identification of specific receptors. These activated receptors recruit LC3s/GABARAPs, a complex at the membrane of the phagophore (a precursor cysterna of the autophagosomes), through an LC3-interacting region, which proceeds with the sequestration of ER fragments into the double-membrane vesicles of autophagosomes, thus delivering the ER for vacuolar/lysosomal clearance (9, 60).

The last 10 years have witnessed the discovery of different ER-phagy receptors, and now, the family of mammalian ER-phagy receptors is large and includes proteins with two reticulon-like homology domains (RHDs), such as FAM134A, FAM134B, FAM134B-2, FAM134C, and RTN3L (61, 62), and a series of others such as SEC62 (63), CCPG1 (64), ATL3 (65), and TEX264 (66, 67). Many ER phagy receptors, such as those of the FAM134 family, contain their LC3-interacting region at the end of a long intrinsically disordered region (LIR). This long region makes the receptor protrude through the ribosome of the rough ER and binds the membrane of the phagophore to promote rough ER clearance (9). Thus, RHD mediates the fragmentation of the ER and the LIR motif sequesters the fragmented ER into autophagosomes.

ER misfolded proteins are degraded by the ER-associated degradation (ERAD) pathways, a complex that leads the recognition of the misfolded polypeptides, their dislocation across the ER membrane, and the retrotranslocation in the cytosol to degrade them by the cytosolic ubiquitin proteasome system. This occurs through retrotranslocation complexes including E3 ubiquitin ligases together with adaptor proteins, which are engaged in a client-specific manner depending on the position of the folding defect, the disulfide bonds, and the N-glycans of the misfolded proteins. ERAD-incompetent proteins segregate in specialized ER subdomains and are eventually cleared by ER-phagy. Thus, in mammals, ER-phagy is a homeostatic mechanism to eliminate misfolded proteins resistant to ERAD (68, 69).

SEC62 selectively delivers an excess of ER generated after ER stress and containing molecular chaperones but not proteins belonging to ERAD, for endolysosomal degradation, thus forming the catabolic pathway of recov-ER-phagy (**Figure 2**) (70).

Differently from ER stress sensors (IRE1, PERK, and ATF6) that contain a luminal domain through which they sense the stress of the organelle, some ER-phagy receptors (FAM134B and RTN3) lack the luminal domain and thus engage adaptors that have domains facing the ER lumen to sense the condition of the ER. For example, FAM134B associates with the lectin chaperone calnexin, which binds ERAD-resistant glycoproteins and segregates them in ER subdomains that may be cleared from cells together with their toxic content by ER-phagy (**Figure 2**) (9, 71, 72). Some pathogens such as Dengue and Zika viruses, have evolved mechanisms to escape ER-phagy by encoding a protease, NS2B3, that cleaves FAM134B, inhibiting its activity and enhancing viral replication, suggesting the mechanisms of ER-phagy inactivation (73).

## Crosstalk between ER-phagy and unfolded protein response

The crosstalk between ER stress/the UPR and ER-phagy is well documented: cell-cycle progression gene 1 (CCPG1) is an ER-resident and a non-canonical cargo receptor that directly binds to core autophagy and thus facilitates ER-phagy. The CCPG1 gene is induced by ER stress and thus directly links ER stress to ER-phagy. Functionally, CCPG1 protects the exocrine pancreas from the consequences of unfolded proteins and proteotoxicity (64).

Pathogen infections induce the UPR together with ER-phagy to promote multiple homeostatic responses for cell survival after infection (74). ER-localized UFMylation, an ubiquitin-like post-translational modification, is required for ER-phagy to repress an IRE1α-mediated unfolded protein response (75). SEC62-mediated recov-ER-phagy is activated upon ER stress to degrade excessive ER and resume ER function (70). All this evidence suggests that ER stress and the consequent UPR is well interconnected with ER-phagy, also through several regulatory steps that, in many cases, favor one of the two while repressing the other.

## ER-phagy and cancer

Here, we will analyze a few examples of the involvement of some ER-phagy receptors in cancer pathogenesis.

FAM134B was overexpressed in esophageal squamous carcinoma (ESCC), and its mutations were detected in cases of ESCC with lymph node metastases (76, 77), suggesting a correlation between the levels of expression/mutations in FAM134B and the aggressiveness of this cancer. The overexpression of FAM134B, quite likely induced by hypoxia, was found in chronic myeloid leukemia (CML) cells and correlated with a poor prognosis (78).

Differently, the small molecule Z36 upregulates FAM134B and causes cell death by generating excessive ER-phagy (79). Recent findings indicate that during hypoxia, FAM134B forms a complex with the ER stress-induced chaperone BIP to target the damaged portions of ER to autophagosomes. Interestingly, the small molecule vitexin, a plant-derived flavone O-glycoside, inhibits ER-phagy by disrupting the FAM134B-BIP complex, together with the ability of BIP to chaperone proteins (**Figure 3**). This double effect of the inhibition of BIP’s folding capacity and of ER-phagy repression suppresses breast cancer growth (80). Thus, it has been suggested that targeting the FAM134-BIP complex may offer a valid strategy to treat cancer.

In contrast, FAM134B loss may promote colorectal cancer tumorigenicity (81) pointing to a tumor suppressor role for FAM134B-mediated ER-phagy, suggesting that targeting this pathway in colorectal cancer is not a good strategy.

High levels of SEC62 are associated with non-small cell lung cancer and thyroid cancer, and silencing SEC62 makes cells more sensitive to ER stress-induced death (82). Likewise, SEC62 amplifications correlate with the highest incidence of high-grade squamous intraepithelial lesions and squamous cell cervical carcinomas, while its silencing inhibits the migration of HeLa cells (83). This suggests that the SEC62-mediated recov-ER-phagy is a homeostatic response to ER stress that might promote cancer survival and migration.

The role of ER-phagy in cancer is still seen as quite complex, and we have no univocal answer whether ER-phagy aids the proliferation and survival of cancer cells or their death. What emerges is the need to pay attention to the delicate balance between ER-phagy, which can help cancer cells to survive, and excessive ER-phagy that instead promotes their death. This mirrors a somewhat-similar scenario of the consequences of ER stress in cancer: moderate ER stress triggers the homeostatic UPR and favors cancer thrive, while unresolved excessive ER stress triggers a maladaptive UPR and cell death (6).

To conclude, ER-phagy receptors may aid or counteract the pathogenesis of different cancers, so their involvement in this process is highly contextual. The thought-provoking question is how to deal with the quantification of ER-phagy in cancers to see whether in certain cancer contexts, it is excessive, and so, pushing it might be detrimental. In this direction, it is envisioned as the strategical analysis for measuring the amount of ER-phagic flux, using tandem fluorescent protein-tagged ER reporters directly in cancer cells (84).

Another important point to help in solving this conundrum between adaptive and maladaptive ER-phagy in cancer is to look at it in relation to the proteostasis. Clearly, some cancer cells, i.e., the highly secretory ones, may not easily deal with defects in proteostasis, so the loss of function of ER-phagy receptors might be not tolerated and the cancer cells succumb. However, more studies are needed to see whether and in what cancer context targeting ER-phagy might be an effective anticancer pharmacological strategy.

## Conclusion

We have described the main modulators of the UPR and ER-phagy, as these might be new targets for impeding cancer growth and spread. The role of the UPR in cancer-relevant processes like angiogenesis, EMT, metastasis, and chemotherapy resistance has been clearly established, as well as the UPR endows cancer cells with greater malignant potential, but the role of ER-phagy in cancer is still controversial. However, initial evidence suggests that the two processes of the UPR and ER-phagy are triggered by common ER stressors and linked, to favor ER homeostasis, while, sometimes, targeting them might trigger cancer death by raising the ER stress levels. The fine-tuned balance in cancer between ER-phagy and excessive ER-phagy might offer the key interpretation to the final output of life versus death in cancer cells, so quantitative measurements of the process are required for rational targeting. Furthermore, given the connection between the PERK and the autophagy pathway in tumors and to study the layers of interconnection and complementarity between the two pathways, we aim to test whether drugs inhibiting the PERK pathway, for example, ISRIB, which has a good safety profile and is effective in restraining the growth of some tumors, can also act on ER-phagy.

## Data availability statement

The original contributions presented in the study are included in the article/supplementary material. Further inquiries can be directed to the corresponding author.

## Author contributions

AC wrote the manuscript, EZ acquired funding and wrote the manuscript. All authors contributed to the article and approved the submitted version.

## Acknowledgments

This study was supported by AIRC MFAG 20018 grant to EZito.

## Conflict of interest

The authors declare that the research was conducted in the absence of any commercial or financial relationships that could be construed as a potential conflict of interest.

## Publisher’s note

All claims expressed in this article are solely those of the authors and do not necessarily represent those of their affiliated organizations, or those of the publisher, the editors and the reviewers. Any product that may be evaluated in this article, or claim that may be made by its manufacturer, is not guaranteed or endorsed by the publisher.
